# A unified model of species abundance, genetic diversity, and functional diversity reveals the mechanisms structuring ecological communities

**DOI:** 10.1111/1755-0998.13514

**Published:** 2021-10-23

**Authors:** Isaac Overcast, Megan Ruffley, James Rosindell, Luke Harmon, Paulo A. V. Borges, Brent C. Emerson, Rampal S. Etienne, Rosemary Gillespie, Henrik Krehenwinkel, D. Luke Mahler, Francois Massol, Christine E. Parent, Jairo Patiño, Ben Peter, Bob Week, Catherine Wagner, Michael J. Hickerson, Andrew Rominger

**Affiliations:** ^1^ Biology Department Graduate Center of the City University of New York New York New York USA; ^2^ Biology Department City College of New York New York New York USA; ^3^ Division of Vertebrate Zoology American Museum of Natural History New York USA; ^4^ Department of Biological Sciences University of Idaho Moscow Idaho USA; ^5^ Institute for Bioinformatics and Evolutionary Studies (IBEST) University of Idaho Moscow Idaho USA; ^6^ Department of Life Sciences Imperial College London Ascot Berkshire UK; ^7^ Centre for Ecology, Evolution and Environmental Changes/Azorean Biodiversity Group Faculdade de Ciências Agrárias e do Ambiente Universidade dos Açores Açores Portugal; ^8^ Island Ecology and Evolution Research Group Institute of Natural Products and Agrobiology IPNA‐CSIC) La Laguna, Tenerife Canary Islands Spain; ^9^ Groningen Institute for Evolutionary Life Sciences University of Groningen Groningen The Netherlands; ^10^ Department of Environmental Science, Policy, and Management University of California Berkeley California USA; ^11^ Department of Biogeography Trier University Trier Germany; ^12^ Department of Ecology and Evolutionary Biology University of Toronto Toronto Ontario Canada; ^13^ CNRS Inserm CHU Lille University of Lille Lille France; ^14^ Center for Infection and Immunity of Lille Institut Pasteur de Lille Lille France; ^15^ CNRS Evo‐Eco‐Paleo SPICI Group University of Lille Lille France; ^16^ Plant Conservation and Biogeography Group Departamento de Botánica Ecología y Fisiología Vegetal Facultad de Ciencias Universidad de La Laguna Tenerife Islas Canarias Spain; ^17^ Group of Genetic Diversity through Space and Time Department of Evolutionary Genetics Max Planck Institute for Evolutionary Anthropology Leipzig Germany; ^18^ Department of Botany and Biodiversity Institute University of Wyoming Laramie Wyoming USA; ^19^ Division of Invertebrate Zoology American Museum of Natural History New York New York USA; ^20^ School of Biology and Ecology University of Maine Orono Maine USA; ^21^ Maine Center for Genetics in the Environment University of Maine Orono Maine USA

**Keywords:** community ecology, community genetic diversity, community phylogenetics, comparative phylogeography, population genetics

## Abstract

Biodiversity accumulates hierarchically by means of ecological and evolutionary processes and feedbacks. Within ecological communities drift, dispersal, speciation, and selection operate simultaneously to shape patterns of biodiversity. Reconciling the relative importance of these is hindered by current models and inference methods, which tend to focus on a subset of processes and their resulting predictions. Here we introduce massive ecoevolutionary synthesis simulations (MESS), a unified mechanistic model of community assembly, rooted in classic island biogeography theory, which makes temporally explicit joint predictions across three biodiversity data axes: (i) species richness and abundances, (ii) population genetic diversities, and (iii) trait variation in a phylogenetic context. Using simulations we demonstrate that each data axis captures information at different timescales, and that integrating these axes enables discriminating among previously unidentifiable community assembly models. MESS is unique in generating predictions of community‐scale genetic diversity, and in characterizing joint patterns of genetic diversity, abundance, and trait values. MESS unlocks the full potential for investigation of biodiversity processes using multidimensional community data including a genetic component, such as might be produced by contemporary eDNA or metabarcoding studies. We combine MESS with supervised machine learning to fit the parameters of the model to real data and infer processes underlying how biodiversity accumulates, using communities of tropical trees, arthropods, and gastropods as case studies that span a range of data availability scenarios, and spatial and taxonomic scales.

## 1 INTRODUCTION

Biodiversity is structured hierarchically across spatial, temporal, and taxonomic scales (Leibold & Chase, 2017). Fluctuations of species abundances within communities operate on ecological timescales, on the scale of handfuls or tens of generations. Population genetic variation, by contrast, accumulates and degrades over timescales of tens to tens of thousands of generations (Leffler et al., 2012), while phylogenetic and functional diversity accumulate even more slowly, on the order of thousands to millions of generations (Uyeda et al., 2011). Over time, various fields have emerged to investigate processes within individual levels of organization (macroecology, comparative population genetics, macroevolution), but only recently have inroads been made to combine theory across multiple scales of space and time into a general unified model (Thompson et al., 2020; Vellend, 2010, 2016). Complicating matters, there is little consensus over whether, and to what degree, ecological interactions contribute to the structuring of ecological communities (Harmon & Harrison, 2015; Rabosky & Hurlbert, 2015). Likewise, the relative contributions of colonization and in situ speciation to the composition of community structure remains an open question (Patino et al., 2017).

Discovering universal rules that structure ecological communities is a challenging task given the difficulty of disentangling the relative influence of faster ecological mechanisms from slower evolutionary processes (Ricklefs, 2004), yet a unification of theory across multiple scales will provide significant insight into the formation of biodiversity (McGill et al., 2019). Ecological models of community biodiversity inspired by the Equilibrium Theory of Island Biogeography (MacArthur & Wilson, 1967) and the Neutral Theory of Biodiversity and Biogeography (Hubbell, 2001) have focused on predicting only a single biodiversity metric, the shape of the local species abundance distribution (SAD). As central as the SAD is to macroecology and community ecology, it is often insufficient to distinguish among different models of community assembly, particularly at equilibrium (Chave et al., 2002; Haegeman & Etienne, 2011; McGill et al., 2007). Recently, DNA sequence data sampled at the community‐scale has offered a powerful new approach for studying community dynamics at the genetic level (Baselga et al., 2013, 2015; Dapporto et al., 2019; Múrria et al., 2017; Papadopoulou et al., 2009). While empirical investigation of community intraspecific genetic diversity has flourished, modelling efforts have remained constrained, with current theory either lacking an explicit population genetic foundation (Vellend, 2005), considering genetic variation only of a focal species (Laroche et al., 2015), or modelling but not fully exploring genetic variation at the community scale (Aguilée et al., 2018; Manceau et al., 2015). Demonstrating the power of unified modeling, a great deal of work has been done to incorporate phylogenetic information with abundance data to make inferences about community assembly processes (Jabot & Chave, 2009; Webb et al., 2002). While such approaches make useful predictions, they are predicated on assumptions of equilibrium within the local community and also assume that the phylogeny is a reliable proxy for functional trait diversity, an assumption violated by traits that are not phylogenetically conserved (Cavender‐Bares et al., 2009). Likewise, there have been other successful efforts to unify theory across timescales with mechanistic ecoevolutionary models of assembly. Cabral et al. (2019) unify population‐level and evolutionary timescales to investigate the dynamic relationship between community age, competition, and local richness. Pontarp et al. (2019) developed a trait‐based, spatially explicit ecoevolutionary model to make inferences about prey and predator niche widths with potentially diverse data types. Incorporating temporal dynamics can help to distinguish among ecological processes (Chisholm & O’Dwyer, 2014; Jabot et al., 2018; Ricklefs, 2006), yet current theory fails to generalize across levels of biological organization. Adding more axes of data to process‐based models without increasing model complexity at the same rate is therefore a necessary advance to break this many‐to‐one mapping of hypotheses to observation (Leibold & Chase, 2017; McGill et al., 2007).

The massive multidimensional data sets that continue to emerge from high‐throughput biodiversity investigations applying community‐wide surveying techniques such as eDNA (Deiner et al., 2017), metabarcoding (Andújar et al.,^2018^; Dopheide et al.,^2019^), and remote‐sensing technologies that can directly infer trait data (Cavender‐Bares et al., 2017), are therefore timely. However, the challenges associated with moving beyond descriptive approaches of interpretation and inference have limited broader understanding of processes generating biodiversity patterns (but see Bohan et al., 2017; Derocles et al., 2018). Historically there have been two general approaches to investigate the evolutionary and assembly processes underlying the patterns we observe in nature: (1) “process‐first” approaches that use first principles to derive generative mechanisms to make predictions of a single data type under the assumptions of an idealized community (Gavrilets & Vose, 2005; Marquet et al., 2014; Rosindell et al., 2012); and (2) “pattern‐first” approaches that reveal aggregate differences in macroecological patterns from real world systems across a range of spatial and temporal scales (Craven et al., 2019; Keil & Chase, 2019; Ricklefs & Bermingham, 2001; Rominger et al., 2016; Wagner et al., 2014). Recent advances in simulation‐based inference under increasingly complex models provide a third option of unifying multiple processes and multiple data types across different scales (Overcast et al., 2019; Pontarp, Bunnefeld, et al., 2019). A unified model of community assembly, which accounts for the fundamental processes underlying biodiversity across spatial and temporal scales (Vellend, 2010), could be used to make predictions about multiple axes of biodiversity data that include species richness and abundances, distributions of species genetic diversities, and trait variation. Several studies have recently shown that such complex biological models and resultant high‐dimensional data can be tractable within a machine learning framework (Schrider & Kern, 2018), providing a robust inference procedure for simulation‐based interrogation of empirical data.

Here we introduce the massive ecoevolutionary synthesis simulations (MESS) model, building upon classic community ecology theory (Hubbell, 2001; Leibold & Chase, 2017; MacArthur & Wilson, 1967; Vellend, 2016) to produce a mechanistic model of local community assembly for making joint predictions of observed multidimensional biodiversity data such as that currently emerging from high‐throughput metabarcoding studies (Taberlet et al., 2012). MESS integrates ecological models of community biodiversity, comparative population genetics, and trait evolution, with an explicit focus on incorporating microevolution and ecological interaction processes, which are often underrepresented in mechanistic models (Leidinger & Cabral, 2017). MESS can simulate community assembly across a continuum of scenarios from evolved to assembled, and from purely neutral to niche‐structured by either competition or environmental filtering. These simulations generate predictions for locally sampled distributions of abundance, genetic variation, and trait values which are summarized using a novel combination of statistics that capture the variation within and among these biodiversity data axes. We combine summary statistics from numerous simulations with supervised machine learning methods to test an array of competing models and to estimate model parameters relevant to understand complex histories of community assembly and evolution. We perform extensive simulation‐based cross‐validation analyses to explore precision and accuracy of model inference. Finally, we apply the model to four high‐throughput biodiversity data sets representing different taxonomic and spatial scales: two arthropod communities with varying dispersal capacity from Mascarene islands of different ages (Emerson et al.,^2017^; Kitson et al., 2018); plot level sampling of Australian tropical forest trees (Rossetto et al., 2015); and archipelago‐scale sampling of Galapagos Islands gastropods (Kraemer et al., 2019; Triantis et al., 2016).

## 2 MATERIALS AND METHODS

### 2.1 Metacommunity composition

**FIGURE 1:**
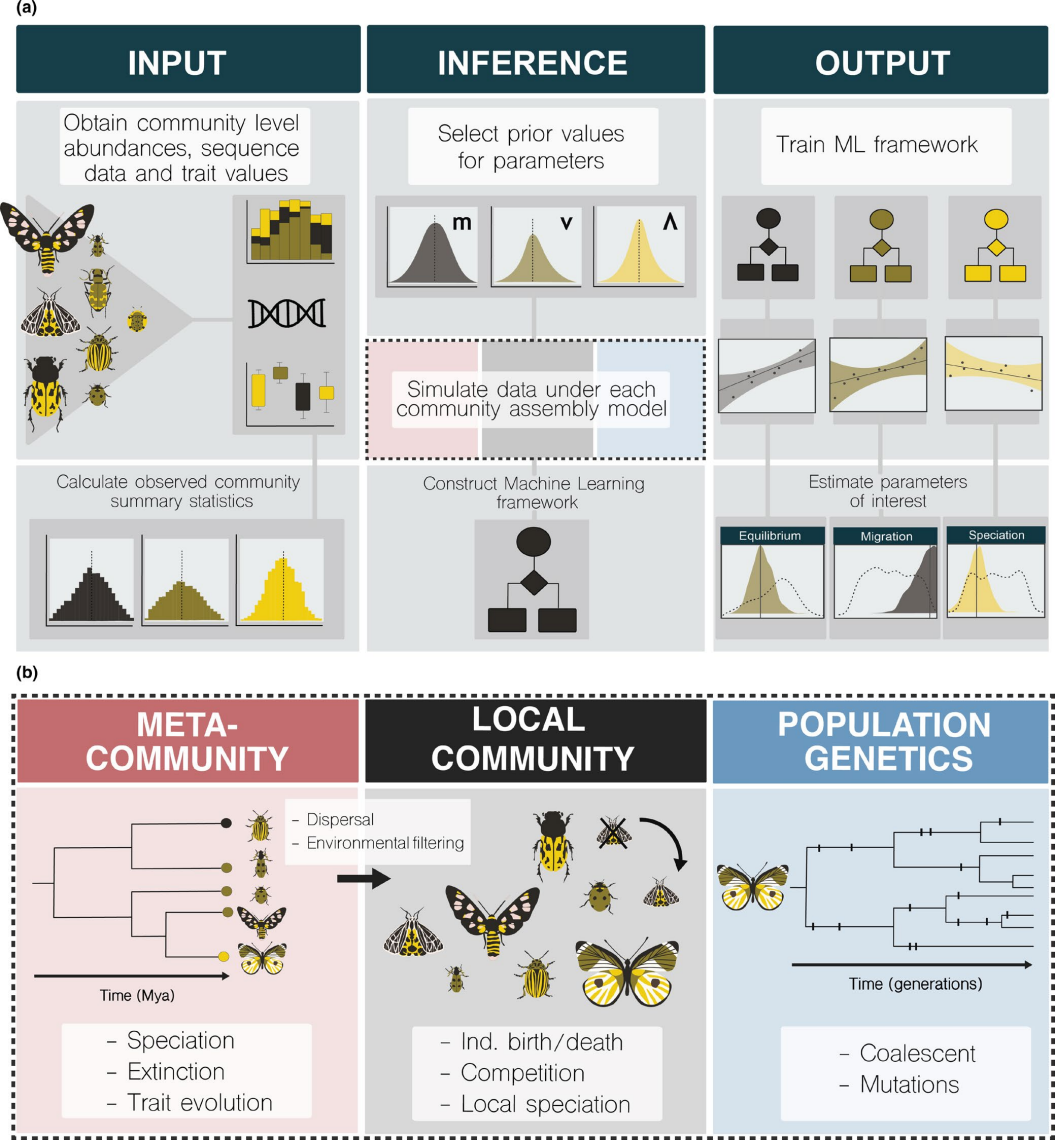
Conceptual diagram illustrating the machine learning inference procedure and the three primary components of MESS simulations. (a) The MESS machine learning inference procedure proceeds broadly in three steps. First, community‐scale data is obtained for one or more axes of biodiversity data including abundances, trait values, and genetic sequence data, and community summary statistics are calculated. Next, prior ranges on model parameters are selected (depicted are migration rate (m), speciation rate (ν), and equilibrium (Λ)), numerous simulations are performed to match the sampling of the observed data using parameters sampled from these prior ranges (dashed box; see exploded view of simulations in (b), and the identical suite of summary statistics are calculated. Finally, a machine learning framework is trained using the simulated data, learning the mapping between summary statistics and simulation parameters. The trained machine learning framework is then used to estimate model parameters using the observed community summary statistics. (b) MESS simulations are composed of three hierarchically linked components. The metacommunity component (red) encompasses a global phylogenetic history of all species, along with species abundances and trait values evolved along the phylogeny. The local community component (black) involves a forward‐time process during which a local community assembles by individual birth/death, immigration (dispersal from the metacommunity), and local speciation or extinction. The population genetic component (blue) approximates per species genetic polymorphism from coalescent simulations that are parameterized from the abundance histories and colonization times generated by the forward‐time local community component. Processes which operate within and between each hierarchical level are indicated within each subpanel (see Figure 2 for further details on model parameters)

**FIGURE 2:**
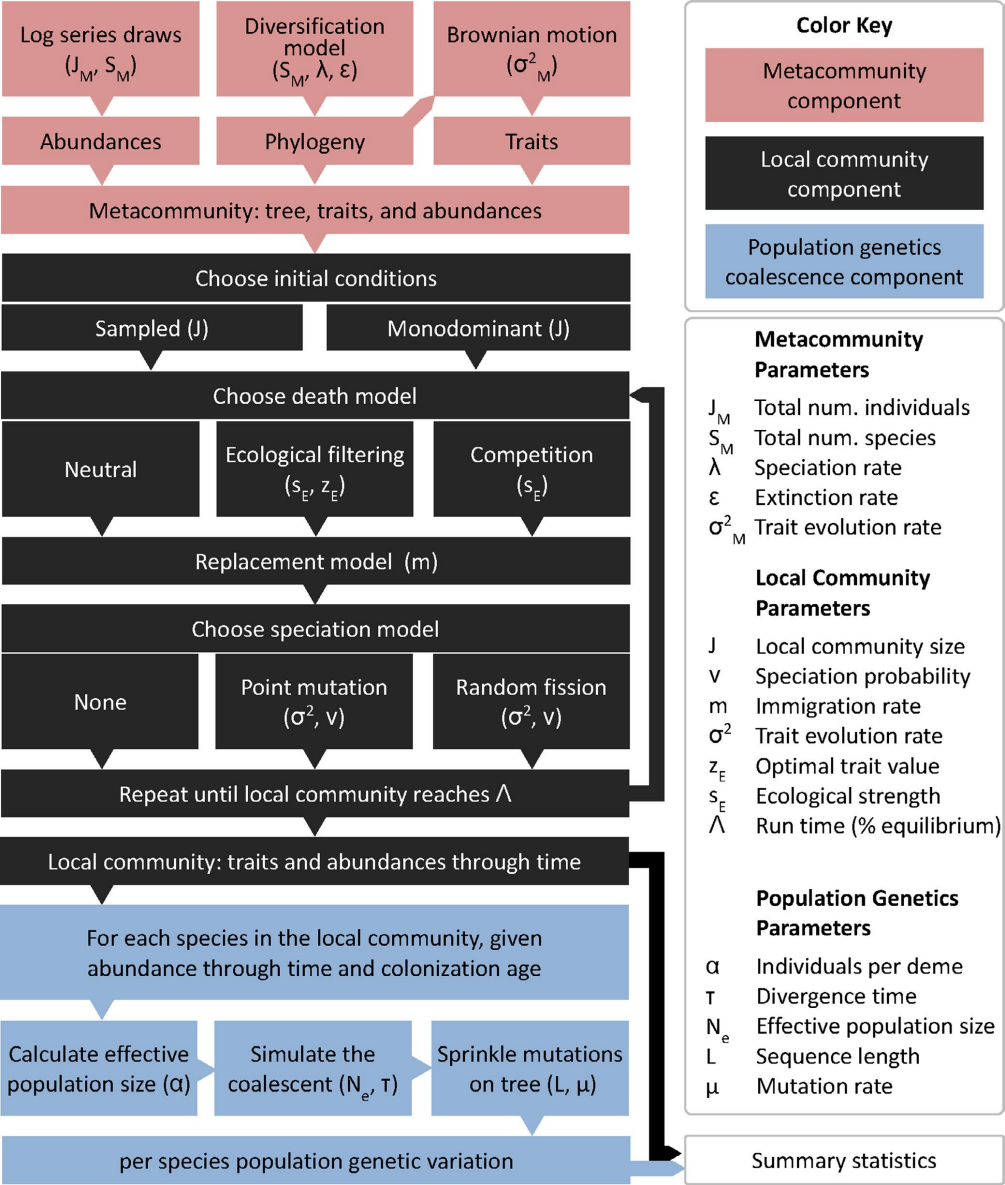
Flow diagram illustrating MESS model processes and parameters. A flow diagram illustrating all MESS model processes and the parameters that govern their behaviour. Each box illustrates a subcomponent of the model (colored to correspond with subcomponents illustrated in Figure 1b), and indicates the parameter(s) which determine the behaviour of each subcomponent. Diversification and trait evolution processes in the metacommunity (red) are determined by speciation (λ) and extinction (ε) rates, the total number of species in the metacommunity (*S_M_*), and the rate of trait evolution (σ^2 M^), which follows a Brownian process. Abundances in the metacommunity are sampled from a log series distribution such that the total number of individuals is equal to J_M_. The local community (black) is initialized with a fixed number of individuals (J) and proceeds by a stepwise birth/death/immigration/speciation process, which (in the neutral case) is governed by the immigration rate (m) and the local speciation probability (ν), and which proceeds for a fixed amount of time per simulation determined by the Λ parameter. For non‐neutral local community dynamics, unequal death probabilities (i.e. fitness differences) are determined by species trait values, the strength of ecological interactions (*s_E_*) and the local trait optimum (*z_E_*; in the case of environmental filtering). Finally, the population genetics component (blue) generates predictions of genetic variation per species based on standard population genetic parameters which are either fixed for all species per simulation (sequence length (L), mutation rate (μ), and number of individuals per deme (*α*)) or which are dynamically recorded per species per simulation (divergence time (*τ*) and effective population size [*N*_e_]). Arrows between subcomponents indicate information flow through the simulations

The MESS model comprises three components summarised in Figures 1b and 2 (See Table 1 for model parameter details). The metacommunity is modelled as a regional pool which is very large and fixed with respect to the timescale of the assembly process in the local community. It consists of a global phylogeny relating all species, along with species abundances, and trait values evolved along the phylogeny. The global phylogeny is produced by simulating a homogeneous, time‐constant diversification process, in which lineages give rise to new lineages or die with fixed speciation (λ) and extinction (λ·ϵ) rates, until the desired number of species ($S_{M}$) is reached (treesim v2.4; Stadler, 2019). Next, we simulate a Brownian motion model of trait evolution on the phylogeny with a root value of 0 and a rate of *σ* ^2 M^ (ape v5.3; Paradis et al., 2004). Continuous traits evolve following a Brownian process of random drift in the metacommunity, rather than an Ornstein–Uhlenbeck process, which is stochastic with a central tendency (Butler & King, 2004), because we assume species in the metacommunity are not exposed to constraints imposed by the local environmental conditions. Likewise with this model, we make no assumption about the degree of phylogenetic conservatism for each trait simulated. While multiple traits evolving under varying degrees of phylogenetic conservatism may provide more nuanced biological insight, for reasons of computational tractability we consider individual trait evolution as a reasonable first approximation. Additionally, we do not model intraspecific trait variation, on the assumption that trait values represent the mean phenotype of each species. Finally, the species abundances are sampled from a log‐series distribution parameterized by the total number of species ($S_{M}$) and the total metacommunity size ($J_{M}$).

**TABLE 1:** MESS model parameters

Categorical parameters	
Parameter	Options
Community assembly model	Neutral/Competition/Environmental filtering
*In situ* speciation model	None/Point mutation/Random fission
Local community initial conditions	Metacommunity sample/Monodominance

Symbol	Meaning of parameter	Type and range
*Metacommunity component parameters*		
*J_M_*	Total number of individuals	Integer ≫ 1
*S_M_*	Total number of species	Integer > 1
Λ	Per lineage birth rate (speciation)	Real in (0,∞)
Ε	Per lineage death rate (extinction) as proportion of λ	Real in [0,1]
$\sigma_{M}^{2}$	Trait evolution rate variance (Brownian motion)	Real >0
*Local community component parameters*		
*J*	Total number of individuals	Integer >1
*S*	Local species richness*	Integer >1
ν	Per capita per birth speciation rate	Real in [0,1]
m	Immigration rate from metacommunity (per step)	Real in [0,1]
*σ* ^2^	Trait evolution rate variance*	Real > 0
*Z_E_*	Optimal trait value in environment*	Real
*S_E_*	Strength of ecological filtering	Real > 0
Λ	Fraction of turnover equilibrium*	Real in [0,1]
*Population genetics coalescence component parameters*		
*L*	Sequence length of simulated genomic region (bp)	Integer > 0
*µ*	Mutation rate	Real > 0
*α*	Abundance/*N*_e_ scaling factor	Integer > 0

Note

All MESS model parameters, their interpretations and range of possible values. Parameters indicated with an asterisk (*) are pseudoparameters which are either emergent, compound, or randomly sampled from a distribution with parameters determined by other elements of the model.

### 2.2 Local community dynamics

The foundations of the community dynamics underlying MESS are based on the joint neutral model of abundance and genetic diversity described in Overcast, et al. (2019). The individual based community assembly model broadly follows that used in Rosindell and Harmon (2013), which is inspired by the ecological neutral theory of Hubbell (2001). A fundamental assumption of this theory is that all species are ecologically equivalent (exchangeable) and that community assembly dynamics are governed entirely by ecological drift. The MESS model implements a birth/death/colonization/speciation process within a semi‐isolated local community of fixed size (*J*) and proceeds in discrete time steps as follows. In each time step an individual is randomly sampled and removed from the local community. Under neutral dynamics all individuals are equally likely to be removed, that is, die, irrespective of their species identity. The sampled individual is immediately replaced by a new individual to maintain constant saturation of the local community. With probability 1‐m, where m is the immigration rate, the replacement is the offspring of another individual in the local community. The local community is assumed to be well mixed, so the parent of the offspring is chosen at random from all individuals in the local community, excluding the recently deceased individual. With probability m the replacement individual is a migrant arriving from the metacommunity. All metacommunity individuals are equally likely to colonize; however, because the species have different metacommunity abundances, not all species are equally likely to colonize. In either case, the new individual inherits the species identity and trait value of its parent. The metacommunity is sufficiently large ($J_{M}\gg J$) such that the species, along with their abundances and trait values, are assumed to remain static with respect to the timescale of assembly in the local community. As with the typical spatially implicit neutral model (Hubbell, 2001), the local community diversity approaches a dynamic equilibrium state from its initial conditions such that ultimately local extinction due to ecological drift is counterbalanced by new species arriving through colonisation.

Departing from the previous model, MESS allows relaxation of the assumption of ecological neutrality, generating individual fitness differences which account for biotic and/or abiotic interactions. MESS local community dynamics can range from fully neutral (species traits have no effect), to various degrees of non‐neutrality determined by the magnitude that species traits influence individual death probability (δ) through competition or environmental filtering. Following Ruffley et al. (2019), we based our environmental filtering and competition models on a functional relationship common in coevolutionary models which relates trait‐based interactions with the probability of persistence in a community, scaled by the ecological strength of the interaction ($s_{E}$; Lande, 1976; Nuismer & Harmon, 2015). The $s_{E}$ parameter determines either the strength of species‐species competitive interactions or species‐environment filtering interactions depending on whether a competition or filtering model is specified. MESS does not simultaneously model competition and filtering, though this will be a potential future development. Calculated death rates per species are normalised to provide a vector of death probabilities that weight the random sampling of which individual will die in each time step according to a multinomial distribution (see Appendix S1:Methods).

As a first approximation, within the local community we implement a point mutation speciation process (Hubbell, 2001), although other modes could be incorporated in future versions of the model (Haegeman & Etienne, 2017; Rosindell et al., 2010). Speciation is implemented phenomenologically and takes place with probability ν upon each birth event. Upon each speciation event, the new individual is assigned a unique species identity, and its prior species identity is recorded as the parental species for purposes of building the local phylogeny. The descendant species receives a trait value sampled from a normal distribution centered on the parent species’ trait value and with variance equal to *σ* ^2 M^/(λ+λ·ϵ), which is the expected variance of trait differences between parent and offspring species in the metacommunity. As each simulation proceeds, trait values continue to evolve in a punctuated fashion at each speciation event, and branch lengths within local radiating lineages are updated to reflect the accumulated time since speciation.

### 2.3 Population genetics component

Following Overcast, et al. (2019), the forward‐time histories of colonization and abundance changes through time per species are rescaled to parameterize divergence time and effective population size in backward‐time coalescent models with immigration for each species (Kelleher et al., 2016) to generate sampled local nucleotide diversities (π; Nei & Li, 1979). For reasons of computational efficiency, and to achieve a realistic scale in terms of numbers of individual organisms, we use a scaling parameter (α) to specify the number of individuals per deme, thus the total number of organisms in the local community is given by J·α. This notion of demes, or ‘cohorts’, groups of individuals that perform the same actions at the same time, is conceptually similar to that of Harfoot et al. (2014). We use the forward‐time frequency of colonization events (scaled to number of colonizations per generation) for each species to parameterize the migration probability in the coalescent of colonization/divergence with ongoing immigration. The per site per generation mutation rate is μ and we use the harmonic mean of the forward‐time population size history of each species to approximate each corresponding effective population size (Karlin, 1968; Pollak, 1983). The time of initial colonization of each species is the divergence time from the source population in the metacommunity within which the final coalescent events take place (going back in time). We scale forward‐time Moran time steps by a factor of 2/J to convert to backward‐time Wright‐Fisher units of nonoverlapping generations. Finally, given an observed data set, coalescent simulations match the observed sample sizes of each species for which DNA sequence data was obtained with regards to numbers of individuals per species and length of sequence.

### 2.4 Summary statistics

We specify a hierarchical structure of summary statistics for each target data axis: species abundances, population genetic variation, and trait values. First, several relevant summary statistics are calculated per species, for each of the data axes. Next, each species‐level statistic is aggregated and community‐scale summary statistics are calculated per axis of data, capturing information about the distribution of the statistic across the community. We include as summaries the first four moments of each community‐wide distribution, as well as pairwise Spearman rank correlations among all data axes. For correlations involving the trait axis, we consider the absolute value of the difference between the species trait and the local trait mean as the trait variable. We also calculate the differences between regional and local values of trait mean and standard deviation ($\Delta_{\mu }^{\mathrm{trait}}$ and $\Delta_{\sigma }^{\mathrm{trait}}$ respectively). Additionally, we utilize a framework of generalized Hill numbers as community‐scale summary statistics, to quantify the shape of each distribution (Chao et al., 2014). In order to distinguish between these diversity metrics when calculated on distributions of different data axes we will refer to the Hill number of order *q* for abundance data as *^q^D*, for genetic data as *^q^GD*, and for trait (functional) data as *^q^FD* (see Appendix S1:Methods for further details). For simplicity, throughout the manuscript we will refer to Hill numbers calculated on distributions of each data axis as abundance, π, and trait Hill numbers.

As an example of the hierarchical nature of our summary statistics, consider genetic variation per species within a local community. The average number of pairwise differences among sampled gene copies (π; Nei & Li, 1979) is calculated to summarize the genetic diversity of each species. As a per species metric π is well suited for characterizing genetic diversity of molecular data as it is able to capture most of the true population genetic diversity with only 5–10 individuals (Tajima, 1983). The per species π values are accumulated to compose the community genetic diversity distribution, and the first four orders of *^q^GD* of this distribution are calculated, summarizing the partitioning of genetic variation at the community scale. A similar hierarchical decomposition of abundance and trait diversity can be obtained. Importantly, with respect to the question of bias induced in summary statistics by unsampled taxa, within the local community it is reasonable to assume that unsampled taxa will be at very low abundance (Preston, 1948). In this case the failure to sample them will have essentially no impact on the abundance, π, and trait Hill numbers, and will induce relatively minor bias in the first four moments, though investigating the nature of this bias is beyond the scope of this manuscript. The complete MESS model predictions are compared with empirical data via summary statistics and machine learning inference methods enabling selection between local community models as well as estimation of parameters relevant to the community assembly process.

### 2.5 Model behaviour

We simulated communities under a range of parameter values to understand how different model processes affect the distributions of community‐scale data, and whether the summary statistics capture information to discriminate among various alternative models. Given that the MESS model is dynamic in time, we controlled for this by running each simulation to the same fixed point in the assembly process. We quantified this point as the proportional approach to equilibrium (Λ) and fixed this parameter at 0.75. This value is measured as the fraction of information about the initial state of the local community which is no longer present in the current state (see Overcast, et al., 2019 for a full treatment of this parameter). We allowed ν to take one of three values corresponding to no‐, low‐ and high‐speciation (0, 5 × 10^−4^, and 5 × 10^−3^ respectively) and generated 10,000 simulations for each assembly model (see Table S1 for simulation parameters). We also investigated how summary statistics of different assembly model types vary through time. To this end, we generated 10,000 simulations for each assembly model while allowing ν to vary as above, sampling communities at different stages of the assembly process (Λ ~ U[0,1]; see Table S2 for simulation parameters).

### 2.6 Machine learning inference and cross‐validation

The MESS package includes an automated multistage machine learning (ML) inference procedure (Figure 1a). First, MESS model parameters of interest are identified for estimation, and prior ranges are established based on some knowledge of the system under investigation. Next, simulations are performed until parameter space is sufficiently sampled. The quantity of simulations to perform depends on the system under investigation and the number of parameters being explored, but 1e5 is on the right order. Prior to ML model training, we perform a feature selection procedure in order to remove all summary statistics that are invariant or uninformative with respect to the target classes (*boruta_py* v0.1.5; Kursa & Rudnicki, 2010). Performance of the ML model hyperparameters (e.g., the number of trees in a random forest and the maximum tree depth) that dictate the structure and functioning of the algorithms. Performance can vary greatly between different data sets and different parameterizations, so MESS tunes these by optimizing cross‐validation scores using a random search method to explore broad priors placed on hyperparameter space. Next a model selection procedure is performed, during which an ML classifier is trained on the simulations using the summary statistics as features and the community assembly model class (Neutral, Filtering, Competition) as the target variable. The trained model is then confronted with empirical data and the predicted model class probabilities are generated. Next, the best community assembly model class is selected as that with the highest predicted probability, and a parameter estimation step is performed. Simulations are filtered to retain only those which belong to the best model, and an ML regressor is trained on this subset of simulations. A second round of feature selection and ML model hyper‐parameter tuning is performed prior to ML regressor model training. Following this, summary statistics from empirical data are used to estimate MESS parameters of interest. We quantify uncertainty on parameter estimates as prediction intervals (PIs) using a quantile regression approach (Meinshausen, 2006). At this stage we are careful to evaluate parameter estimate uncertainty in light of the fact that uncertainty on model selection has not been propagated forward, which is an avenue for further development. Finally, to evaluate model adequacy we implement posterior predictive simulations (PPS) to assess goodness of fit of the model to the observed data (Gelman, 2003). Additionally, after both classification and regression training steps, feature importances can be extracted to evaluate the proportion of information with respect to a given target variable that is contained within each retained summary statistic. The MESS ML classification and regression procedures can be performed with a number of ensemble learning strategies including random forest (Breiman, 2001), gradient boosting (Friedman, 2001), and adaboost (Freund & Schapire, 1997). Unless otherwise indicated, all ML algorithms are implemented in python using the architecture of scikit‐learn (v0.20.3; Pedregosa et al., 2011).

We explored the power, accuracy, and bias of the ML inference procedure to classify community assembly models and estimate parameters using simulation experiments and cross‐validation (CV). To evaluate assembly model classification, we generated 10,000 simulations per model class (i.e., neutral/filtering/competition) and fixed all MESS parameters at intermediate values, varying only the size of the local community (*J*) and the local speciation probability (v) (see Table S3 for simulation parameters). To quantify the accuracy and bias of MESS parameter estimation utilizing an ML ensemble method regression framework, we generated 10,000 community simulations per assembly model class while varying several parameters of interest (α, *J*, *s_E_*, *m*, v, and Λ) using log‐uniform or uniform prior distributions (see Table S4 for parameters). ML estimator performance was then investigated using a K‐fold CV procedure whereby simulations were split into training and testing sets, with the model being iteratively trained on each K‐fold and performance being evaluated as minimized CV prediction error on the held out training set. Classifier model adequacy was quantified by the percent error rate of misclassification, and regression model accuracy was quantified by the explained variance and R^2^ (coefficient of determination) regression scores.

### 2.7 Empirical examples

As case studies, we selected four systems that occupy different spatial scales and probably occupy different locations on the continua of dispersal, speciation, ecological drift and non‐neutrality. Each system has some combination of community‐scale data available for two of the three axes which can be considered by the model. In this way we hope to demonstrate the power of MESS across taxonomic and spatial scales, using data availability scenarios that might be encountered by empirical biologists in the present or very near future. These systems are: (1) spiders from Réunion island with abundances collected from ten 50 × 50 m plots and 1,282 individuals sequenced for one ~500 bp mtDNA region (COI; Emerson et al., 2017); (2) weevils from two Mascarene islands (Réunion and Mauritius) which have been densely sampled for abundance and sequenced for one mtDNA region (~600 bp COI) at the community‐scale (Kitson et al., 2018); (3) three subtropical rain forest tree communities scored for multiple continuous traits and shotgun sequenced for whole cpDNA (Rossetto et al., 2015); and (4) Galapagos snail communities collected from all major islands, sampled for one mtDNA region (~500 bp COI; Kraemer et al., 2019) and scored for two continuous traits (Triantis et al., 2016). For both the tree and snail communities, we collapsed the multidimensional trait data using principal component (PC) analysis, and selected the position of each species along PC1 as its trait value. For each empirical data set we conducted 10,000 simulations of each assembly model class and generated abundances, trait values, and genetic variation corresponding to genomic regions with identical numbers of base pairs under an infinite‐sites model at a rate sufficient to generate diversity similar to the empirical data (see Appendix S1: Methods for precise empirical data curation and simulation procedures). We then conducted a round of ML model selection, parameter estimation, and quantile regression to generate parameter estimates and PIs. Finally, we implemented PPS to assess goodness of fit of the selected model and parameters to each of the observed data sets. For the PPS we generated 100 simulations using the estimated MESS model parameters and reduced the resulting simulated and observed summary statistics into lower‐dimensional space by applying principal component analysis. We assessed centrality of the empirical summary statistics in PC space with respect to simulated summary statistics to evaluate goodness of fit.

## 3 RESULTS

### 3.1 Model behaviour

**FIGURE 3:**
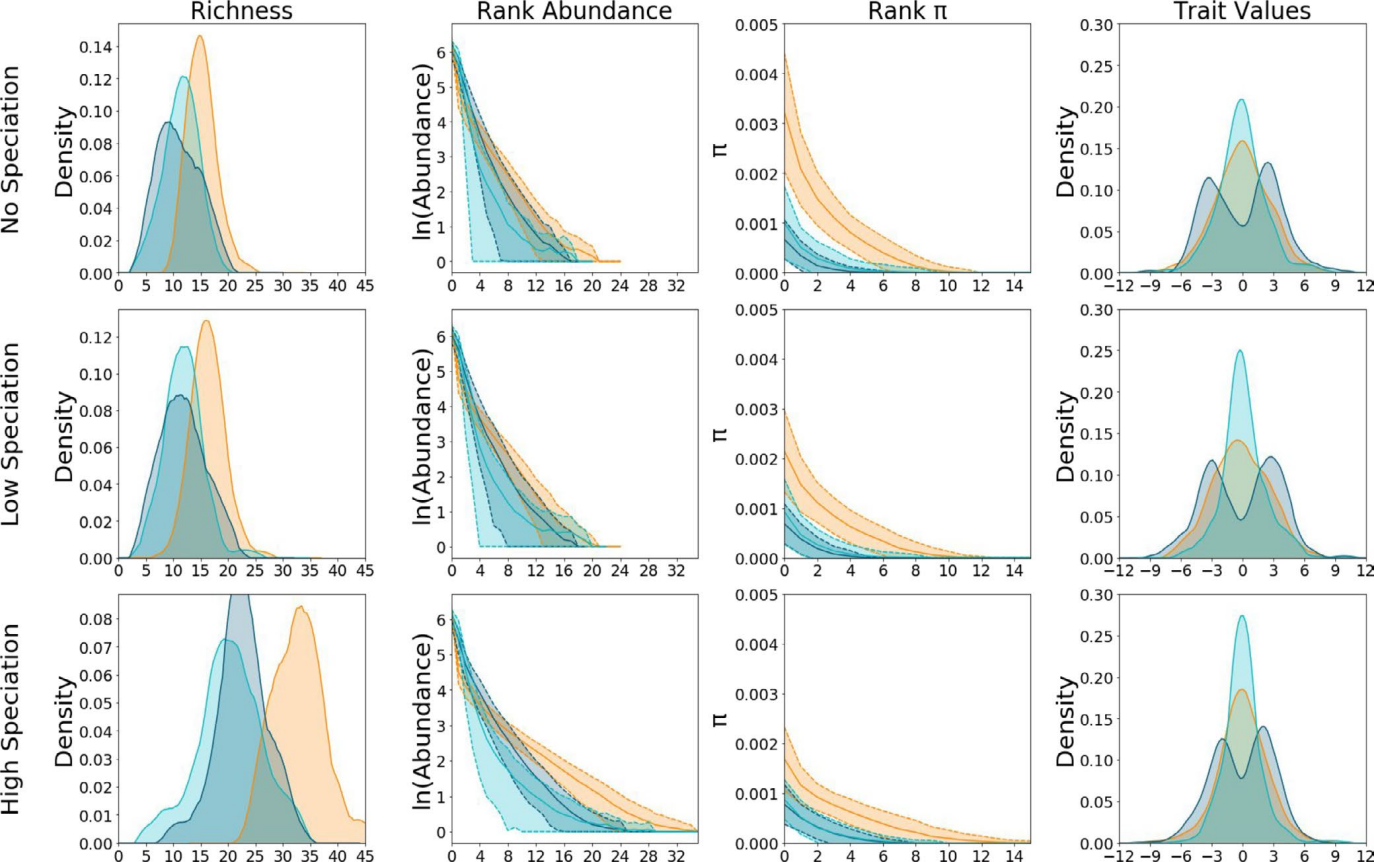
Effect of varying speciation rate and community assembly model on summary statistics. Species richness, rank abundance, rank genetic diversity, and rank distributions for 1000 simulations generated under neutral (orange), competition (dark blue) and filtering (aqua) scenarios with time fixed at 500 generations. From bottom to top, rows of panels correspond to simulations with high (ν = 0.005), low (ν = 0.0005) and no (ν = 0) speciation. In the left column of panels, kernel density plots indicate the distribution of richness across simulations. In the rank plots (centre two columns of panels), thick lines indicate average rank values and shaded areas show plus and minus one standard deviation. The right column of panels shows kernel density plots of zero‐centred trait distributions

Simulations generated under different community assembly models produced markedly different distributions of community‐scale data and summary statistics. First we considered one static point in time (at Λ = 0.75; Figure 3). Neutral simulations generated communities with higher species richness, more even distributions of abundance as summarized by the normalized *^q^D* values, and higher mean and standard deviation of π values. Filtering and competition models were largely indistinguishable in terms of abundance and genetic diversity, with distributions of species richness, and mean and standard deviation of the population genetic statistics broadly overlapping (Figure 3). Distributions of statistics related to trait values showed more nuanced and variable behaviour, obtaining characteristics that differ between the three models. There was little distinction between models in terms of distributions of difference in local and metacommunity mean trait values ($\Delta_{\mu }^{\mathrm{trait}}$), with the exception that filtering models produced more variable results. However, distributions of local and metacommunity difference in trait standard deviation ($\Delta_{\sigma }^{\mathrm{trait}}$) varied considerably among models, with competition tending to yield negative values (more variation locally than in the metacommunity), filtering producing positive values (less variation locally in the metacommunity), and neutral models producing values centred on zero. This pattern is borne out in Figure 3, which illustrates the standard deviations of trait values increasing with competition, and decreasing with filtering, with respect to neutral models. The trait diversity values (*^q^FD*) tended to be slightly higher for neutral models.

**FIGURE 4:**
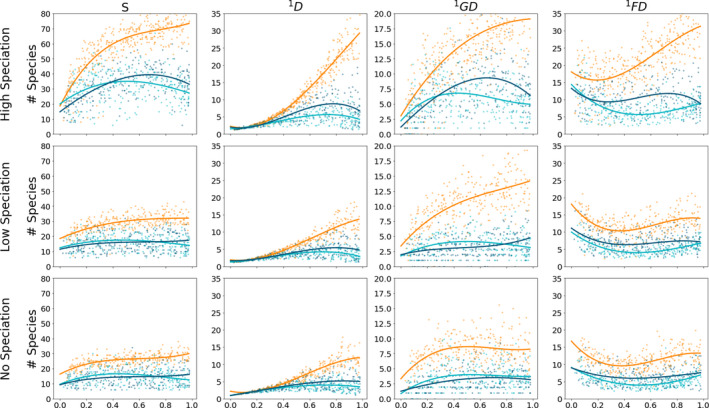
Community summary statistics through time for neutral and non‐neutral models. This plot depicts the temporal change in select summary statistics for the three focal community assembly models at three different speciation rates: No, Low, and High corresponding to ν = 0, 0.0005, 0.005, respectively. The x‐axis indicates community age measured as progress of the community toward equilibrium (Λ). Community assembly models depicted are neutral (orange), filtering (aqua), and competition (dark blue). Each subpanel shows the resultant summary statistic for 1000 simulations equally spaced through time for each model class. Simulated values are depicted as points, and a least squares polynomial is fit to better illustrate the trajectory. The far left column of panels illustrate species richness on the *y*‐axes (S). The y‐axes of the remaining columns illustrate the Hill number of order 1 (effective number of species) for abundance, genetic diversity, and trait values, respectively

Next, we investigated the temporal dynamics of MESS community histories (Figure 4). Again, species richness in neutral models tended to exceed that of the non‐neutral models throughout the entire community assembly process. In general, a low rate of local speciation produced a slight increase in richness and Hill numbers for neutral simulations, whereas a high rate produced dramatic increases in these metrics for all simulation scenarios. Between non‐neutral models, richness and Hill numbers for competition were, on average, always greater than those of filtering models across all time points, with differences increasing with increasing speciation rate (v). For neutral models, *^q^D* tended to slowly increase monotonically through time, whereas *^q^GD* initially increased quickly with community‐scale genetic diversity accumulating more slowly in later stages of assembly. Increasing v increased the average maximum *^q^GD* for non‐neutral models, but in these simulations this maximum value tended to saturate very early, with little change through time. *^q^FD* demonstrated a more dynamic temporal trajectory. Broadly, the relationships among the trait Hill numbers mirrored those of the abundance and π Hill numbers, with neutral models obtaining the highest, filtering the lowest, and competition somewhat intermediate values, and a trend of increasing values through time. However, one key difference in *^q^FD* is that early‐stage communities display relatively high values, with values decreasing as Λ increases from 0 to ~0.2, and then showing an increasing trend as Λ proceeds from 0.2 to 1.

### 3.2 Model selection ML cross‐validation

**FIGURE 5:**
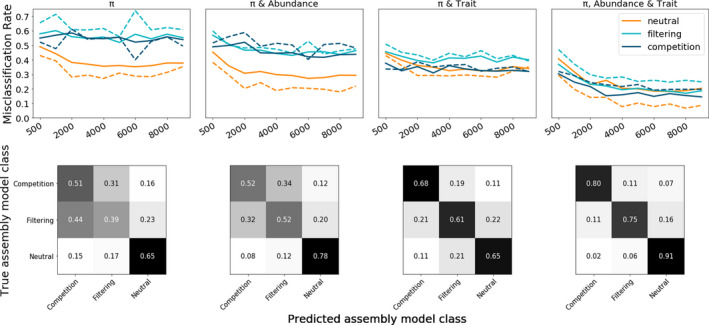
Machine learning classification error rates and confusion matrices. The top row shows random‐forest misclassification error rates given different combinations of available data axes for varying sizes of local communities (J). Data axes used for each suite of simulations are indicated along the top of the figure. The x‐axis indicates increasing sizes of J, from 500–10,000 in regular intervals. The y‐axis indicates probability of assembly model misclassification, averaged over 1000 simulations per model class for each J (i.e., lower values indicate more accurate classification). In the figure, orange shows neutral simulations, aqua shows filtering, and dark blue shows competition. Solid lines indicate 1 ‐ precision and dashed lines indicate 1 ‐ recall. The bottom row shows confusion matrices depicting detailed model misclassification rates for data availability scenarios given J values between 9000 and 10,000. In these figures, values on the diagonals indicate the proportion of accurately classified simulations for each model class. Off‐diagonal values indicate misclassified simulations

ML model classification prediction error reached a minimum value with local community size (*J*) of 10,000 for all model classes and all evaluated feature sets (Figure 5; mean error rate 0.16). Prediction error was slightly higher for small *J* (mean error rate 0.19), and did not improve dramatically when increasing *J* from 1000 to 2000 (mean change in error rate −0.02). Neutral simulations were more accurately classified than non‐neutral simulations across all feature sets and v values (mean error rate 0.05 and 0.18 respectively). ML classifiers trained using summary statistics from all data axes were most accurate; however, including trait information along with just one other data axis (either π or abundance) produced classification error rates close to models trained on the full suite of summary statistics. ML classifiers trained using only summary statistics related to abundance and π produced accurate classification of neutral simulations (mean error rate 0.05), but failed to distinguish between the two non‐neutral models (error rate >0.4). Importantly, in this condition the predicted model class for non‐neutral simulations was overwhelmingly the alternative non‐neutral model and rarely the neutral model. For example, simulations under a competition model were misclassified as filtering (0.35) with a much higher rate than neutral (0.08).

### 3.3 Parameter estimation ML cross‐validation

**FIGURE 6:**
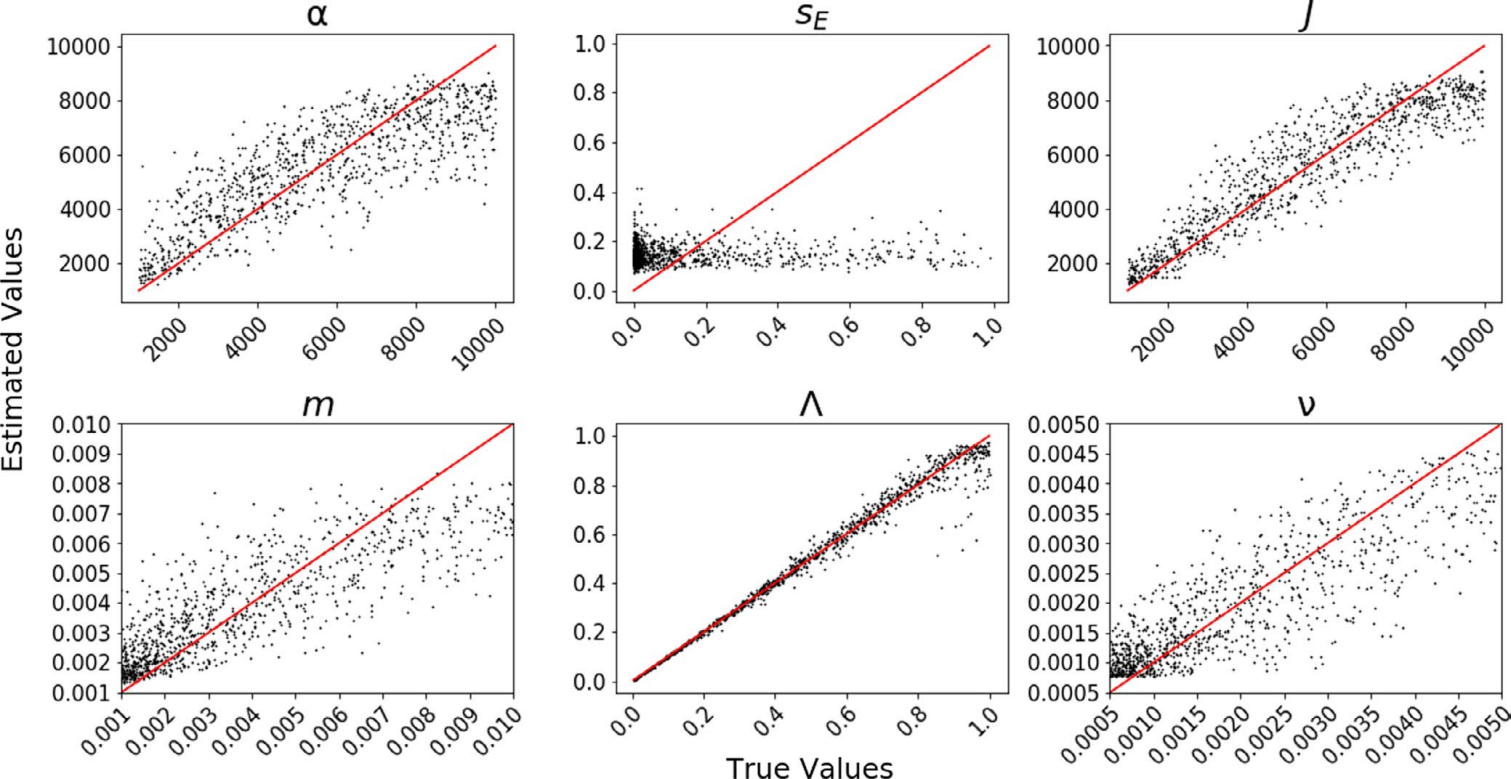
Machine learning cross‐validation parameter estimation. 1000 parameter estimation cross‐validation (CV) replicates using neutral community assembly model simulations and summary statistics from all data axes. True parameter values are on the x‐axes and the corresponding point estimates are on the y‐axes (*R* ^2^ values: α = 0.567, *J* = 0.845, *s_E_* = −0.037, *m* = 0.685, v = 0.714, Λ = 0.963). A parameter that is well estimated will have CV results that fall on or around the identity line (depicted in red). Note that ecological strength (*s_E_*) has no impact on neutral simulations, which produces the poor CV performance in estimating this parameter

Cross‐validation explained variance and R^2^ regression scores for model parameter (α, *J*, *s_E_*, *m*, v, and Λ) estimation were broadly congruent and positive in almost all cases, indicating that the simulated and estimated parameter values were correlated (in some cases highly so). For neutral simulations Λ had the highest *R* ^2^ (0.963) and ecological strength (*s_E_*) the lowest (–0.037), with most parameters having moderate *R* ^2^ values (e.g. *α* = 0.567; *m* = 0.685; Figure 6). The small *R* ^2^ for *s_E_* is expected given that neutral simulations should have no information about strength of environmental interactions. Estimates of small to moderate values of *m* and v were accurate, but larger values tended to be underestimated. ML parameter estimation for simulations of filtering and competition models obtained improved accuracy to estimate *s_E_* (*R* ^2^ = 0.146 and *R* ^2^ = 0.287, respectively); however, *R* ^2^ values for other parameters were somewhat reduced with respect to the neutral simulations (Figures S1 & S2). Both non‐neutral models produced diffuse estimates of α (*R* ^2^ = 0.205 and *R* ^2^ = 0.258) and *J* (*R* ^2^ = 0.398 and *R* ^2^ = 0.448). The most significant difference between the non‐neutral models concerned estimates of Λ. Under competition scenarios, Λ estimates were precise but upwardly biased between Λ = 0 and 0.5, with increasing variance between Λ = 0.75 and 1. Under filtering scenarios, Λ estimates were only accurate for values close to Λ = 0.5, with decreasing accuracy as Λ moved away from this value in either direction.

### 3.4 Empirical examples

**FIGURE 7:**
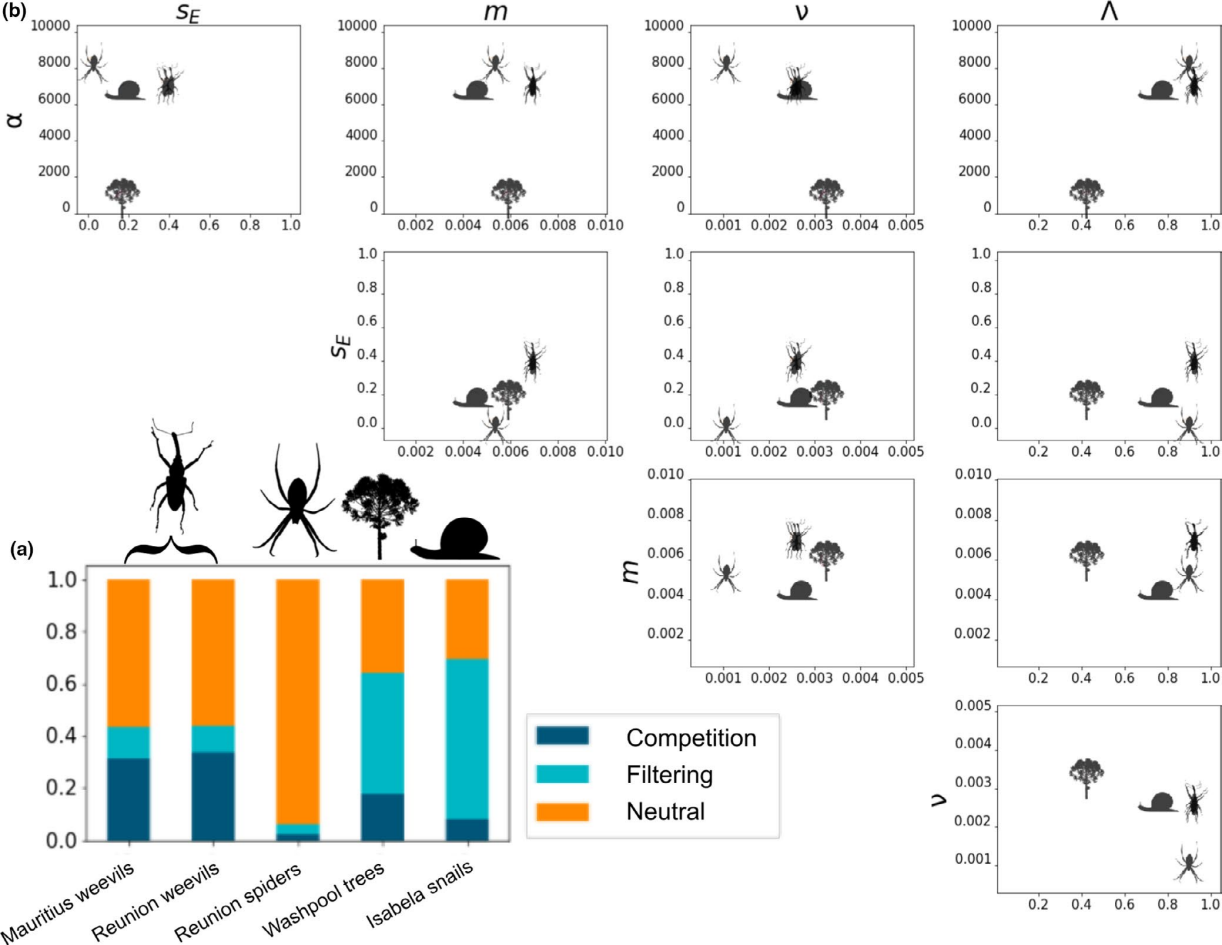
MESS empirical analysis. Empirical classification and parameter estimation of five local communities including snails, tropical trees, and island arthropods. (a) depicts machine learning classification probabilities for each empirical community for three focal community assembly models. The proportion of colour within each bar represents the proportional predicted model class for neutrality (orange), environmental filtering (aqua), and competition (dark blue). (b) depicts pairwise estimates of five different model parameters under the best classified model for each local community data set. The value along each parameter axis is indicated by the position of the representative icon. Parameters depicted include number of individuals per deme (*α*), ecological strength (*s_E_*), migration rate (*m*), local speciation probability (ν), and fraction of equilibrium (Λ)

The ML classification procedure identified the neutral model as the most probable for all three Mascarene arthropod communities (Figure 7a), with considerable support for neutrality of the Reunion spider community (predicted class probability 0.939), and more equivocal class probabilities for Mauritius and Réunion weevil communities (0.566 and 0.53, respectively). The most important features for classification were *^1^D*, standard deviation and mean of π, *^2^D*, and *^4^D* (accounting for 44% of relative importance of all retained features). ML classification identified environmental filtering as the most probable model for all tree and snail communities, with highest support for the snails (mean predicted class probability 0.698), and weaker support for the trees (mean probability 0.440). Combining filtering and competition predicted class probabilities indicated the average probability of non‐neutrality for the trees was 0.633, and for the snails was 0.865. Feature importance values for classification using axes of trait and genetic data were broadly diffuse across the retained summary statistics, with $\Delta_{\sigma }^{\mathrm{trait}}$ accounting for 11% of relative importance of all retained features, and the remainder accounting for 5% or less.

The ML regression procedure for parameter estimation indicated that the selected empirical data sets occupied a broad swath of parameter space (Figure 7b; Table S6). Empirical PIs were quite varied, with some parameter estimate PIs spanning the width of the prior, while the PI of other parameters were narrow, a result which is consistent with CV results. The tree communities had small *α* estimates with narrow PIs (mean *α* = 1423; 1019–2481 95% PI), when compared to the arthropod and snail communities, which had larger *α* estimates (e.g., Mauritius weevil *α* = 7107; 3497–9831 95% PI). ML estimates of Λ were more varied, with the weevil and spider communities approaching or reaching Λ = 1, snail communities having more intermediate Λ, and tree communities having the lowest values (<0.4 in all cases). Estimates of *m* and ν displayed an idiosyncratic pattern, with spider and snail communities having low estimated values for both, weevils having high estimated values for both, and trees having high ν and low *m* estimates. Consistent with the CV experiments, ecological strength (*s_E_*) was the most difficult parameter to estimate, in the sense that all estimates were close to the mean of the prior, and PIs spanned the majority of the prior range. Posterior predictive simulations indicated a good fit of the estimated parameters to all empirical data sets, with the exception of the Reunion spiders (Figure S3).

## 4 DISCUSSION

Community ecology has been perceived as a "mess" (Lawton, 1999) because of the endless proliferation of processes proposed for shaping biodiversity. To remedy this, Vellend (2010) proposed a conceptual framework to unify the study of community assembly dynamics composed of four fundamental processes: dispersal, stochastic drift, selection (e.g., deterministic competition/filtering), and speciation. These different processes operate on different timescales and contribute information to different biodiversity data axes (i.e., species richness, abundances, trait distributions). To more fully characterize the interaction among these processes it is therefore necessary to develop process based joint models of these data axes. Recently, Overcast, et al. (2019) took a new step in this direction and proposed a unified model of community ecology and population genetics, which accounted for local processes of dispersal and drift, and introduced a novel population genetic process. This model makes predictions of local genetic diversity which are a record of community history on an intermediate timescale, and are complementary to joint predictions of abundance. Furthering this unification, here we have described an individual‐based mechanistic model of community assembly, the MESS model, which fully unifies the key processes underlying the dynamics of local accumulation of biodiversity across multiple timescales: dispersal, stochastic drift, selection (e.g., deterministic competition/filtering), and speciation (Vellend, 2010, 2016). The MESS model integrates these processes in an hierarchical framework to make temporally explicit multidimensional predictions of species abundances, population genetic diversities, and trait variation in a phylogenetic context. MESS allows for simulating community‐scale data from communities assembled entirely by in situ speciation (e.g., Galapagos finches; Grant & Grant, 2011) to those assembled only by dispersal (e.g., nearctic snakes; Burbrink et al., 2015), as well as the full continuum between these. Additionally, MESS can generate predictions across the full spectrum of ecological interactions, from complete neutrality to strong niche‐structuring through biotic or abiotic interactions. MESS expands the toolbox of practicing community ecologists by allowing to incorporate community genetic sequence data, along with abundances, and trait data for inferring the processes which have shaped observed biodiversity patterns.

Simulation experiments show that MESS model summary statistics retain a very strong signal of temporal state (Figure 5; Figures S1, S2; Λ subpanels) and that neutral models have elevated *S*, *^q^D*, *^q^FD*, and *^q^GD* compared to filtering and competition models across all except the earliest time points (Figure 4). This is a direct result of the ecological equivalence of individuals in neutral models generating communities with lower species dominance. In a similar fashion, for non‐neutral models, species that are more fit survive preferentially and increase in abundance, reducing evenness in the community and causing ^1^ *D* to plateau at a low level, though it should be noted MESS does not implement negative density dependence and this is an avenue for future research. The finding that neutral models generate the highest species richness may be in conflict with theory that suggests competition is important for maintenance of biodiversity (Tilman, 1994), however the spatially implicit model of competition implemented in MESS may not fully capture competition dynamics, and so this result should be interpreted with care. Increased speciation rate has little impact on ^1^ *D* in the neutral case because ecological equivalence confers no cost or benefit to offspring species, whereas in non‐neutral models new species inherit ancestral trait values with small perturbation. In these conditions increasing speciation rate increasingly favours the evolution and accumulation of small clades of species that have ecological advantage, causing a concurrent reduction in ^1^ *D*.

Overall, we find that any two of the three data axes are sufficient to accurately identify the relative strength of neutral versus non‐neutral processes in local community assembly, and that including trait information allows discrimination between which of the non‐neutral processes are more important in driving the local patterns of biodiversity (Figure 5). This latter finding suggests that niche‐structured abundances and genetic diversity distributions are broadly similar between environmental filtering and competition models, and that the variance in local traits is necessary to distinguish between them. These results should be robust to values of *s_E_* that generate moderate to strong non‐neutrality (i.e. *s_E_* ≥ 1), with a corresponding increase in misclassification rate as *s_E_* approaches 0. More generally, using any two data axes always resulted in improved classification accuracy when compared to using a single axis alone. Furthermore, our results highlight the flexibility of MESS to mask unobserved summary statistics such that inference can be made from a wide variety of high‐throughput biodiversity surveys across different spatial scales and data availabilities. This will enable practicing community ecologists to perform inference with whatever biodiversity data is in hand.

The empirical communities we chose to evaluate represent both a variety of available data axes, and a range of perceived dispersal limitation, with Galapagos snails being the most dispersal‐limited, the Australian trees being least limited, and the Mascarene spiders and weevils somewhat intermediate. The results from the Reunion spider community (classified as neutral with Λ approaching 1, *m* high and *v* low) are consistent with a late‐stage community that is structured primarily by colonization and ecological drift (Vergnon et al., 2012); however, we note that the model provided a relatively poor fit to this data, so this finding should be interpreted with caution. Both weevil communities had similarly high estimates of Λ, but higher estimated *v*, and less clear support for classification as neutrally evolving. The snail communities were classified as being structured by environmental filtering, with low estimated *m* aligning with expectations of low dispersal. However, the low estimates of *v* and *s_E_* are somewhat surprising, given their documented pattern of single‐island endemism (Parent & Crespi, 2006). Finally, because the Australian tree communities are plot‐level samples from smaller scales representing semi‐isolated habitat patches and not true insular systems we expect their parameter estimates to deviate from those of true island assemblages. This is in agreement with the finding that these tree communities are all far from equilibrium (Rossetto et al., 2015). Specifically, our approach estimates that the system is characterized by moderate *m*, and high *v* and *s_E_* estimates which indicate that local turnover, in the context of a selective environment, is important and ongoing.

### 4.1 Future perspectives

As a first approximation of the feedbacks between processes operating at different timescales MESS makes several simplifying assumptions which can be treated as targets for future model improvement. Non‐neutral dynamics could constrain trait evolution as a function of resource availability or density‐dependence (Múrria et al., 2018), allow for filtering and competition processes within the same model, and/or allow for mutualistic rather than simply competitive interactions. Additionally, directly modelling multivariate trait evolution may increase statistical power of inference (Zheng et al., 2009) while bypassing the biases associated with reducing the dimensionality of multivariate data into one trait dimension (e.g., with PCA; Uyeda et al.,^2015^). Modelling more realistic metacommunity processes and patterns, and including more sophisticated measures of diversity such as temporal correlations and environmental matching would allow for expanding beyond the simple local/regional dichotomy. One caveat is that MESS assumes all species (or operational taxonomic units) have been well identified and do not deviate from panmictic population structure, as this will distort model selection and parameter estimation during inference. For example, cryptic population structure will reduce *S* and inflate metrics of genetic diversity, which could bias MESS inference to prefer non‐neutral models, within which these features are common hallmarks. Another special consideration is the variance in the rate at which Λ changes with respect to time as measured in generations. Specifically, the neutral approach to equilibrium is much slower (with respect to numbers of generations) than either of the non‐neutral models, potentially confounding comparisons between models at fixed values of Λ. This also highlights the need for a more robust measure of equilibrium, which can account for processes across timescales. From a practical perspective, the limitations of current MESS ML inference (i.e., point estimates of model parameters and uncertainty estimated using quantile regression) may be overcome by implementing a machine learning procedure which would allow for full posterior inference (e.g., Bayesian additive regression trees; Chipman et al., 2010).

Another approximation is the use of the rescaled Wright‐Fisher coalescent process to generate the community‐wide population genetic predictions of the forward‐time Moran birth/death process. Yet future advances could make use of the powerful new tree‐sequence recording (Haller & Messer, 2019; Kelleher et al., 2018) to more accurately and flexibly match the full demographic and abundance history of each species with its respective underlying population genetic history. Although here we modelled a single locus per species to match the barcode and metabarcode data that are emerging from high‐throughput ecological sampling efforts, implementing tree‐sequence recording methods could also allow for flexible downstream options to incorporate spatial information associated with genetic georeference databases (Lawrence et al., 2019).

### 4.2 Conclusions

With our approach we were able to identify whether real communities were near equilibrium or not, and the ecoevolutionary processes underlying those dynamics. For example, despite the near‐equilibrium state of both spider and beetle communities on islands, we discovered that the approach to these equilibria were different, with spider communities assembling largely by immigration, compared to the more prominent role of speciation in weevil communities. This confirms suspected, but as of yet untested, hypotheses from other island arthropod systems (Rominger et al., 2016) that can only now be evaluated. We were also able to pinpoint the mechanistic causes (turnover and environmental filtering) of nonequilibrium in the tree communities. Finally, our analysis of Galapagos snails highlight areas for future improvement in modeling more fine scale environmental heterogeneity and its impact on filtering and speciation.

The MESS model unifies the study of biodiversity by linking ecological and evolutionary theory across three disparate timescales within an individual‐based, mechanistic framework. The model generates explicit temporal predictions of community‐scale data across these three diversity axes (species richness and abundance, population genetic diversity, and trait variation), spanning equilibrium and nonequilibrium conditions, and allowing for stochasticity along a continuum of scenarios ranging from pure ecological neutrality, to strong ecological interactions and/or environmental filtering. To complement the MESS model simulations, our implementation includes an extensive suite of ML tools for performing model selection and parameter estimation from observed data, and plotting routines for visualizing and evaluating results. This unified mechanistic model provides a general framework for hypothesis testing and biodiversity data synthesis, enabling the generation of multidimensional forecasts and test parameterized hypotheses about the historical and future processes driving biodiversity patterns from small‐scale intensively sampled plots, to islands sensu lato, to regional and subcontinental scales.

## AUTHOR CONTRIBUTIONS

Isaac Overcast, Megan Ruffley, James Rosindell, Luke Harmon, Michael J. Hickerson and Andrew Rominger designed the model and wrote the first draft of the manuscript. Isaac Overcast, Megan Ruffley and Andrew Rominger implemented the model and conducted all simulations and analyses. All authors contributed substantially to manuscript revisions and development of the conceptual framework.

## Supporting information

Appendix S1

## Data Availability

The MESS model software and all jupyter notebooks sufficient to reproduce the simulations, analyses, and figures of this manuscript have been made available in the GitHub repository: https://github.com/messDiv/MESS. No new data were used in this manuscript, and all empirical data that were analysed are available in the aforementioned GitHub repository.
