# Supplementary material for: A unified model of species abundance, genetic diversity, and functional diversity reveals the mechanisms structuring ecological communities
Source: Mol Ecol Resour. 2021 Oct 23;21(8):2782–800. doi: 10.1111/1755-0998.13514 (PMC9297962; doi:10.1111/1755-0998.13514)
Supplement: Supplementary file 1 — Appendix S1 [file MEN-21-2782-s001.pdf]

A unified model of species abundance, genetic diversity, and functional diversity reveals the mechanisms structuring ecological communities

Isaac Overcast*, Megan Ruffley*, James Rosindell, Luke Harmon, Paulo A. V. Borges, Brent C. Emerson, Rampal S. Etienne, Rosemary Gillespie, Henrik Krehenwinkel, D. Luke Mahler, Francois Massol, Christine E. Parent, Jairo Patiño, Ben Peter, Bob Week, Catherine Wagner, Michael J. Hickerson**, Andrew Rominger**

Supporting Methods

Local community initial conditions

The local community has a fixed, finite carrying capacity (J) and is assumed to always be saturated. This is known as the zero-sum assumption and relaxing it typically has no effect on predictions of species abundance within neutral models (Etienne *et al.* 2007). For reasons of computational efficiency, to achieve a realistic scale in terms of numbers of individual organisms, the units used in the local community simulations do not correspond to individual organisms, but rather to demes (or ‘cohorts’, groups of individuals that perform the same actions at the same time, see Harfoot *et al.* 2014). This notion of a deme is conceptually similar to that of a propagule from MacArthur and Wilson (1963), which they defined as "the minimum number of individuals of a given species needed to achieve colonization". We use a scaling parameter α to give the number of individuals per deme and thus the local community size parameter J gives the number of demes in the local community. The total number of organisms in the local community is therefore given by $J \cdot \alpha$. The local community is initialised with individual demes from one or more species chosen from the metacommunity. If the local community represents a continental island, or habitat patch that is well connected to the metacommunity, it is initialised with J independent random samples (with replacement) from the individuals in the metacommunity. Here we are modelling a community of panmictic individuals that are simultaneously and instantaneously isolated from the metacommunity as the initial state. More abundant species in the metacommunity are thus likely to also be more abundant in these initial conditions. Alternatively, if the local community represents a volcanic island origin, or other region of empty habitat (e.g. following a large scale disturbance event), it is initialised as saturated by one species from the metacommunity (Rosindell & Harmon 2013), here we choose the most abundant.

Local community non-neutral dynamics

We based our environmental filtering model on a functional relationship common in coevolutionary models as a way to relate trait interactions between species and their environment with the probability of persistence in a community (Lande 1976; Nuismer & Harmon 2015; Andreazzi *et al.* 2017). Following Ruffley et al. (2019) we calculate the death rate for species i as

$$\delta_i = 1 - exp\left[- \frac{1}{s_E} \left(z_i - z_E \right)^2 \right]$$ (Eq 1)

Here, the species trait is given by z_i, the local community environment has trait optimum z_E, and the strength of the environmental filtering is controlled by s_E. When s_E is small ($\ll 1$), filtering has only a mild effect, individual fitness differences are indistinguishable (i.e. approximately equal δ for all individuals), and the assembly process approaches neutrality. Conversely when s_E is large ($\gg 1$), the filtering effect is very strong, individuals are heavily penalized for traits dissimilar to the optimum (individual δ_i values can vary by several orders of magnitude), and the assembly process is strikingly non-neutral. For intermediate values of s_E the distribution of δ across the community will gradually become more uneven as s_E increases. In general, an s_E of 1 or greater produces a noticeably non-neutral process, with values << 1 approaching neutrality.

We similarly compute the non-neutral death rate due to competitive exclusion such that the probability of an individual dying increases as its trait value approaches the mean trait value within the local community:

$$\delta_i = exp\left[- s_E \left(z_i - \bar{z} \right)^2 \right]$$ (Eq 2)

Here, the local community mean trait is given by $\bar{z}$ and the strength of competition between individuals is given by s_E, all other parameters are as in Equation 1. When s_E is large, competition has a strong effect, individuals with trait values closer to the local mean will have higher δ and proportionally higher probability of death. By contrast, small s_E produces weak competition, a more even distribution of δ, and an increasingly neutral assembly process as s_E approaches 0.

Summary statistics

We utilize a framework of generalized Hill numbers as community-scale summary statistics, following a growing literature indicating their effectiveness for summarizing community-scale patterns in various types of biological data (Chao *et al.* 2014; but see Leinster and Cobbold 2012 for an alternative framework). The attribute diversity component of generalized Hill numbers have the form:

$$^qAD = \left[\sum_{u \in S} v_u \left(\frac{a_u}{\sum_{h \in S} v_h a_h} \right)^q \right]^{1/(1-q)}$$ (Eq 3)

where S is species richness, v_u is the attribute value for species u, a_u is the abundance of species u, and q is the order of the equation. ^qAD quantifies the relative frequency of species attribute values (e.g. abundance, trait, or π) and is undefined for order 1, but a limit exists as q approaches 1 (see Chao et al. 2014). The ^qAD value is difficult to interpret directly and is not

comparable across different data types, but it can be converted into an effective number of species or species equivalents:

$$ {}^{q}D = \left[\frac{{}^{q}AD\left(\sum_{u \in S} v_u a_u\right)}{\sum_{u \in S} v_u a_u} \right]^{1/\varphi} \qquad \text{(Eq 4)} $$

where φ= 1 for species diversity and genetic diversity, and φ= 2 for trait diversity. Hill numbers calculated in this way are not directly comparable across simulations, as different *S* will change their interpretation. To account for this, all Hill number values for all data types are additionally normalized by dividing by *S*, converting them to percentages and allowing for comparability across communities of differing richness.

Simulation parameters and prior ranges

When simulating data with the goal of performing inference on empirical data it is of great importance to choose parameter values and prior ranges on parameters that are informed by knowledge of the focal community. One consideration is that increasing the number of free parameters will exponentially increase the number of simulations that will need to be generated, so it is critical to identify and fix as many parameters as possible to biologically realistic values. A similar concern arises around prior ranges, in that broad prior ranges will require more simulations, while narrow ranges may place undue restrictions on the simulations. In general, metacommunity parameters should be fixed for specific values and not assigned prior ranges, as these will be very difficult to estimate from the data, in practice. In terms of metacommunity parameters, the number of species in the metacommunity (S_M) should be bounded on the low end by the number of species in the focal community, and on the high end by the number of species in the region that could reasonably colonize the local community. If published speciation (λ), extinction (ε) and trait evolution (σ^2_M) values exist, these should be used. If not then preliminary macroevolutionary simulations may be run with external tools, such as ToyTree (Eaton 2020), to identify reasonable values. The number of individuals in the metacommunity (J_M) should be "large" with respect to the abundances of the taxa in the focal community. For mammals 1e5 or 1e6 may be appropriate, whereas for arthropods this should be significantly higher. A precise value for J_M may be difficult to reasonably estimate, but fortunately this parameter is relatively insensitive to misspecification, as it primarily modulates colonization probability. Of the population genetic parameters, only the number of individuals per deme (α) may take a prior range, whereas the mutation rate (μ) and sequence length (*L*) should be fixed to correspond to the values of the data in hand. In terms of a prior range, α is a scaling parameter from demes to individuals to parameterize N_e in the coalescent model. Taking an estimate of θ, for example nucleotide diversity (π), and (for haploid data) rearranging: $\theta = 2N_e\mu$, one may obtain $\theta/2\mu = N_e$ as an estimate of N_e. Dividing this by proposed values of α should be on the order of the average number of samples per population. Parameters dictating processes in the local community are of utmost importance (*J*, *m*, and s_E). The number of individuals in the local community (*J*) may be fixed to the value of the number of individuals in the sample or it may take a prior range to account for sampling bias, values of which should not exceed one

order of magnitude of the number of observed samples. The migration rate into the local community (*m*) will dramatically impact the local community structure. If values are too low (<0.001), the community will approach mono-dominance, and if they are too high (>0.1) the local community will appear as a random sample of the metacommunity. Finally, values of ecological strength (s_E) which are very large (>10) or very small (<0.001) will converge to a neutral model. Therefore, for non-neutral simulations, a reasonable prior range for this parameter is [0.01-5].

Simulation and inference performance and runtimes

The runtimes for any given simulation are a complex function of the input parameters. Simulation runtimes are generally insensitive to metacommunity parameters such as the number of individuals (J_M), the number of species (S_M) and the speciation (λ), extinction (ε) and trait evolution (σ^2_M) rates, as the metacommunity is generated once at simulation initialization, and then remains static for the duration. Local community parameters have a much stronger impact on runtimes, specifically increasing the number of individuals in the local community (*J*) will increase runtimes polynomially in all cases. Decreasing the rate of migration into the local community (*m*) will increase runtimes only when measuring time to equilibrium (Λ). As an example, with all other parameters set to the defaults, running a local community to 100 generations with *J* = 1000 takes ~5 seconds, and with *J* = 5000 takes ~1 minute. Runtimes are less sensitive to population genetic parameters, for example the number of individuals per deme (α) and the mutation rate (μ) will have little impact on runtime for reasonable values, and runtimes will scale linearly with sequence length (*L*). One critical strength of MESS is that it has massive parallelization built-in, automatically scaling to as many processors as are available. Running 10,000 simulations for parameter values used in the simulation experiments (Table S2) on a workstation with 40 cores takes approximately 20 hours.

In terms of fitting MESS to empirical data, runtimes for the built-in automated machine learning (ML) process will vary as a function of the number of simulations in the dataset and chosen parameters of the inference procedure, with the vast majority of this time dedicated to training the ML. Running ML classification and prediction with default parameters will take less than an hour on a standard workstation, though the results will be unsatisfactory. In all cases inference will improve, but runtimes will be greatly increased (on the order of several hours) by including automatic feature selection (`select_features=True`) and ML parameter tuning (`param_search=True`). Once trained MESS ML models may be reused to infer assembly model class and estimate parameters, in which case classification and prediction are functionally instantaneous.

References

Andreazzi, C. S., Thompson, J. N., & Guimarães Jr, P. R. (2017). Network structure and selection asymmetry drive coevolution in species-rich antagonistic interactions. *The American Naturalist*, 190(1), 99-115.

Chao, A., Chiu, C.-H., & Jost, L. (2014). Unifying Species Diversity, Phylogenetic Diversity, Functional Diversity, and Related Similarity and Differentiation Measures Through Hill Numbers. *Annual Review of Ecology, Evolution, and Systematics*. https://doi.org/10.1146/annurev-ecolsys-120213-091540

Eaton, D. A. (2020). Toytree: A minimalist tree visualization and manipulation library for Python. *Methods in Ecology and Evolution*, 11(1), 187-191.

Etienne, R. S., Alonso, D., & McKane, A. J. (2007). The zero-sum assumption in neutral biodiversity theory. *Journal of Theoretical Biology*, *248*(3), 522–536.

Harfoot, M. B. J., Newbold, T., Tittensor, D. P., Emmott, S., Hutton, J., Lyutsarev, V., … Purves, D. W. (2014). Emergent global patterns of ecosystem structure and function from a mechanistic general ecosystem model. *PLoS Biology*, *12*(4), e1001841.

Lande, R. (1976). Natural selection and random genetic drift in phenotypic evolution. *Evolution*, 30(2), 314-334.

Leinster, T., & Cobbold, C. A. (2012). Measuring diversity: the importance of species similarity. *Ecology*, 93(3), 477-489.

Nuismer, S. L., & Harmon, L. J. (2015). Predicting rates of interspecific interaction from phylogenetic trees. *Ecology letters*, 18(1), 17-27.

Overcast, I., Emerson, B. C., & Hickerson, M. J. (2019). An integrated model of population genetics and community ecology. *Journal of biogeography*, 46(4), 816-829.

Rosindell, J., & Harmon, L. J. (2013). A unified model of species immigration, extinction and abundance on islands. *Journal of Biogeography*, 40(6), 1107-1118.

Ruffley, M., Peterson, K., Week, B., Tank, D. C., Harmon, L. J. (2019) Identifying Models of Trait-Mediated Community Assembly Using Random Forests and Approximate Bayesian Computation. *Ecology and Evolution*, 9:13218–13230.

Supporting Tables and Figures

Table S1: MESS model behavior with fixed Λ exploratory analysis simulation parameter values

MESS model parameters used for generating figure 2 in the main text. All parameters are fixed at intermediate values, and only the community assembly model is allowed to vary. The *U*([x]) notation indicates that the exact values within the square brackets were sampled uniformly.

**Parameter**	**Value(s)**
Community assembly model	*U*([Neutral, Competition, Environmental filtering])
In situ speciation model	Point mutation
Local community initial conditions	Monodominance
J_M	5e5
S_M	250
λ	2
ε	0.7
σ^2_M	2
J	1000
ν	[0, 0.0005, 0.005]
m	*0.005*
s_E	*0.1*
Λ	0.75
L	570
μ	2.2e-8
α	2000

Table S2: MESS model temporal behavior exploratory analysis simulation parameter values

MESS model parameters used for generating figure 3 in the main text. All parameters are fixed at intermediate values, and only the community assembly model is allowed to vary. The *U*([x]) notation indicates that the exact values within the square brackets were sampled uniformly.

**Parameter**	**Value(s)**
Community assembly model	*U*([Neutral, Competition, Environmental filtering])
In situ speciation model	Point mutation
Local community initial conditions	Monodominance
J_M	5e5
S_M	250
λ	2
ε	0.7
σ^2_M	2
J	1000
ν	U([0, 0.0005, 0.005])
m	*0.005*
s_E	*0.1*
Λ	*U*(0, 1)
L	570
μ	2.2e-8
α	2000

Table S3: Machine learning classifier model selection simulation parameter values
MESS model parameter values used for model selection simulations and machine learning classifier cross-validation. Parameters with specified ranges were sampled from either uniform (U) or log-uniform(LU) distributions, and all other parameters were fixed at the indicated values. The *U*([x]) notation indicates that the exact values within the square brackets were sampled uniformly.

**Parameter**	**Value(s)**
Community assembly model	*U*([Neutral, Competition, Environmental filtering])
In situ speciation model	Point mutation
Local community initial conditions	Monodominance
J_M	5e5
S_M	250
λ	2
ε	0.7
σ^2_M	2
J	*U*(1000, 5000)
ν	*LU*(0.0005, 0.005)
m	*0.005*
s_E	*0.1*
Λ	*U*(0, 1)
L	570
μ	2.2e-8
α	2000

Table S4: Machine learning regression parameter estimation simulation parameter values
MESS model parameter values used for parameter estimation simulations and machine learning regression cross-validation. Parameters with specified ranges were sampled from either uniform (*U*) or log-uniform(*LU*) distributions, and all other parameters were fixed at the indicated values. The *U*([x]) notation indicates that the exact values within the square brackets were sampled uniformly.

**Parameter**	**Value(s)**
Community assembly model	Neutral
In situ speciation model	Point mutation
Local community initial conditions	Monodominance
J_M	5e5
S_M	250
λ	2
ε	0.7
σ^2_M	2
J	*U*(1000, 5000)
ν	*LU*(0.0005, 0.005)
m	*U*(0.001, 0.01)
s_E	*LU*(0.01, 10)
Λ	*U*(0, 1)
L	570
μ	2.2e-8
α	*U*(1000, 10000)

Table S5: MESS parameter estimates for empirical datasets

Parameter estimates and 95% prediction intervals for the empirical datasets analyzed in the main text. Parameters estimated include number of individuals per deme (α), ecological strength (s_E), migration rate (m), local speciation probability (v), and fraction of equilibrium (Λ).

	**Λ**	**α**	***s_E***	**generation**	**m**	**v**
**Mauritius Weevils**	0.934 (0.804-1.000)	7107.251 (3496.731-9831.389)	0.391 (0.002-0.995)	1259.572 (402.725-3590.325)	0.007 (0.002-0.010)	0.003 (0.001-0.005)
**Reunion Weevils**	0.930 (0.802-1.000)	7178.214 (3506.795-9823.661)	0.415 (0.002-0.997)	1259.572 (402.725-3590.325)	0.007 (0.002-0.010)	0.003 (0.001-0.005)
**Reunion Spiders**	0.894 (0.838-0.999)	8296.900 (6268.000-9735.000)	0.023 (0.001-0.061)	791.100 (392.000-884.000)	0.005 (0.002-0.008)	0.001 (0.001-0.003)
**Australian Trees**	0.426 (0.056-0.834)	855.300 (519.000-1333.000)	0.168 (0.004-0.639)	113.400 (12.000-198.000)	0.006 (0.001-0.010)	0.003 (0.001-0.005)
**Galapagos Snails**	0.758 (0.321-0.995)	6777.409 (1741.969-9876.601)	0.180 (0.001-0.932)	658.921 (110.059-2009.187)	0.004 (0.001-0.009)	0.003 (0.001-0.005)

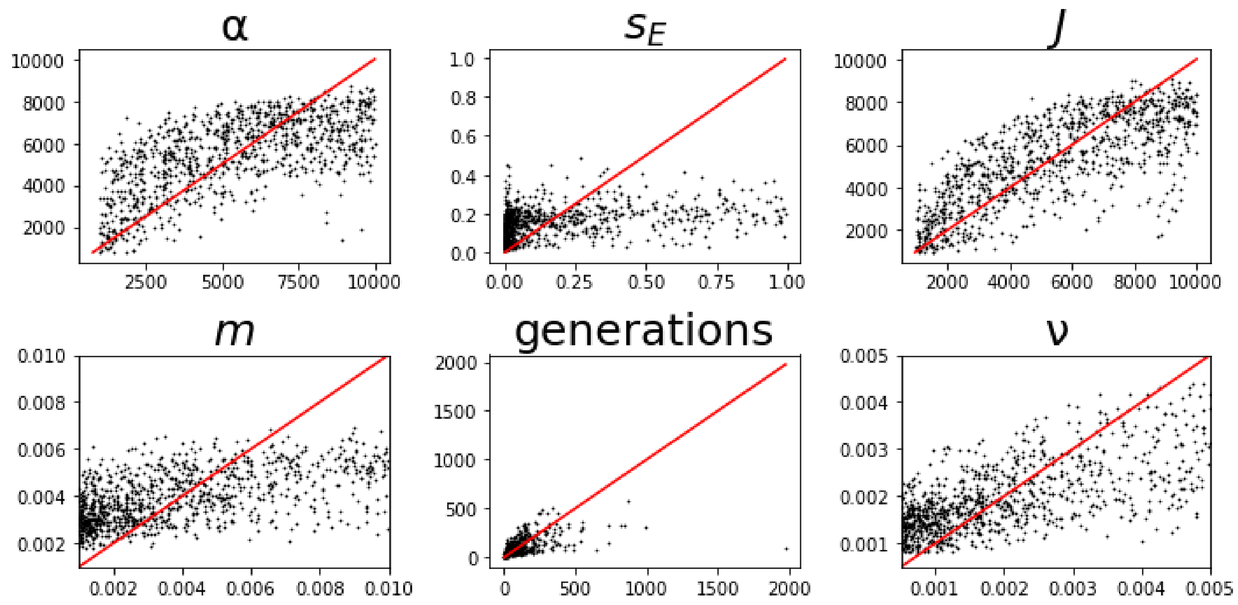

Figure S1: Machine learning cross-validation parameter estimation
1000 parameter estimation cross-validation (CV) replicates using environmental filtering community assembly model simulations and summary statistics from all data axes. True parameter values are on the x-axes and the corresponding point estimates are on the y-axes. A parameter that is well estimated will have CV results that fall on or around the identity line. Parameters depicted are: individuals per deme (α), environmental strength (s_E), local community size (J), migration rate (m), number of generations, and speciation rate (v).

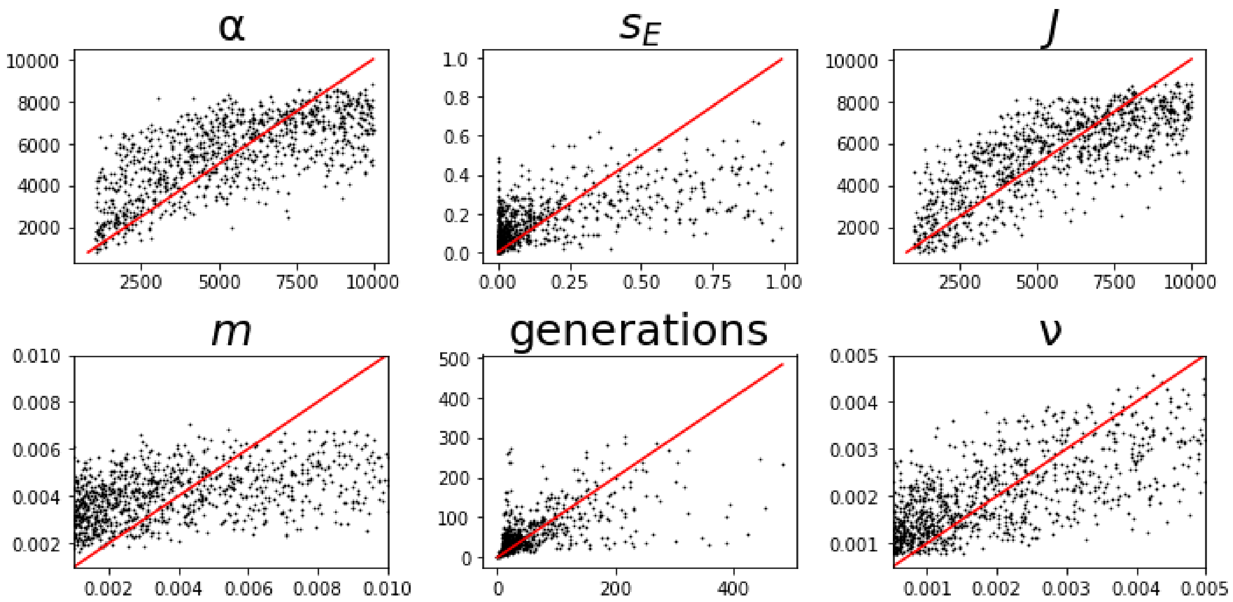

Figure S2: Machine learning cross-validation parameter estimation
1000 parameter estimation cross-validation (CV) replicates using competition community assembly model simulations and summary statistics from all data axes. True parameter values are on the x-axes and the corresponding point estimates are on the y-axes. A parameter that is well estimated will have CV results that fall on or around the identity line. Parameters depicted are: individuals per deme (α), environmental strength (s_E), local community size (J), migration rate (m), number of generations, and speciation rate (ν).

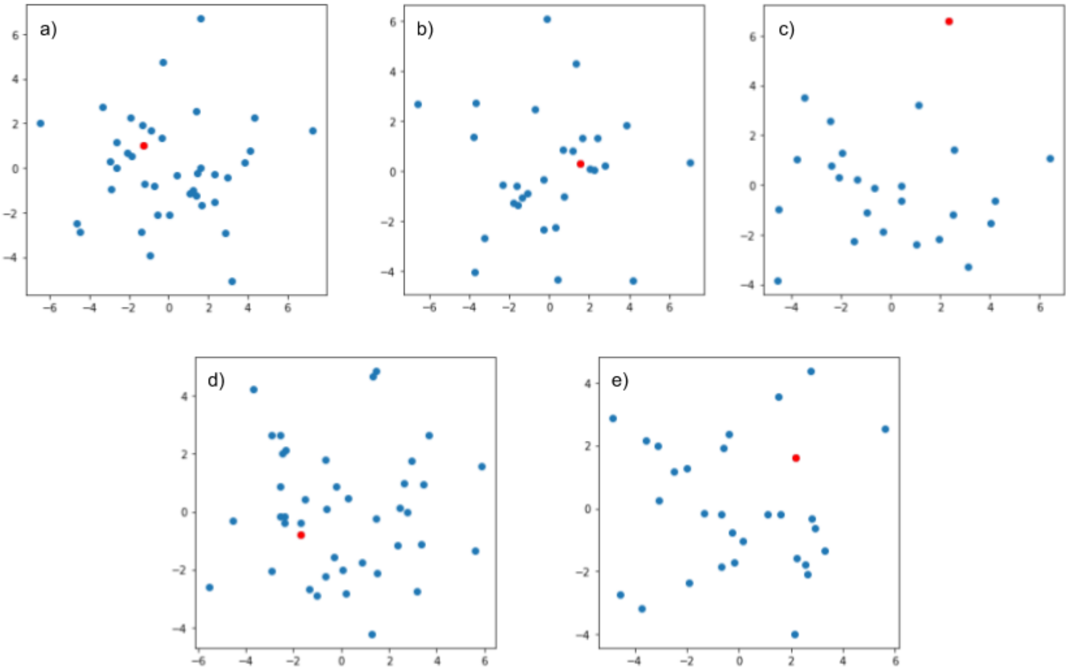

Figure S3: Posterior predictive simulations for the 5 examined empirical datasets
Principal component analysis plots showing summary statistics for 50 posterior predictive simulations (blue points) projected to the first two PCs. Summary statistics of the observed communities are depicted in red: a) Mauritius weevils; b) Reunion weevils; c) Reunion spiders; d) Australian rainforest trees; e) Galapagos snails.
